# Machine learning-based prediction of treatment outcomes and quantitative analysis of contributing factors in fertility induction therapy for adolescent male patients with congenital hypogonadotropic hypogonadism

**DOI:** 10.3389/fmolb.2026.1828462

**Published:** 2026-05-01

**Authors:** Xinyu Dou, Qin Zhang, Yi Wang, Zheng Yuan, Chunxiu Gong

**Affiliations:** Department of Endocrinology, Genetics and Metabolism, Beijing Children’s Hospital, Capital Medical University, National Center for Children’s Health, Beijing, China

**Keywords:** adolescent males, congenital hypogonadotropic hypogonadism, feature importance, machine learning, predictive model, random forest, treatment outcomes

## Abstract

**Objective:**

Congenital Hypogonadotropic Hypogonadism (CHH) is a rare disease with an extremely low incidence, and the outcomes of fertility induction therapy in CHH patients exhibit significant interindividual heterogeneity. Given the context of scarce samples and heterogeneous phenotypes, conventional statistical methods struggle to integrate multidimensional data and quantify the contribution of influencing factors. In contrast, machine learning (ML) techniques offer unique advantages in integrating high-dimensional complex medical data and uncovering hidden relationships. To date, the application of ML for predicting treatment outcomes in CHH remains unexplored. Therefore, this study aims to, for the first time, utilize an ML algorithm to construct and validate a predictive model based on a limited clinical cohort and thereby provide a basis for the individualized treatment of CHH.

**Methods:**

In this single-center retrospective cohort study, 65 adolescent male CHH patients undergoing fertility induction therapy were enrolled and categorized into success (nocturnal emission, n = 55) and failure (non-ejaculation, n = 10) groups based on treatment outcomes. Fifteen pre-treatment baseline indicators across four categories were collected. A random forest model was constructed, employing the Synthetic Minority Over-sampling Technique (SMOTE) and 5-fold stratified cross-validation to mitigate class imbalance and overfitting. Key predictors were identified via feature importance ranking, and decision thresholds were optimized using ROC curves. The model’s performance was comprehensively evaluated and compared against other ML methods.

**Results:**

The random forest model demonstrated excellent and stable predictive performance: Accuracy 0.84 ± 0.12, Precision 0.82 ± 0.13, Recall 0.89 ± 0.08, F1 score 0.85 ± 0.10, and AUC 0.95 ± 0.04. Feature importance analysis identified the top five predictors: cryptorchidism (the strongest predictor), pre-treatment penile diameter, penile length, follicle-stimulating hormone (FSH) level, and anti-Müllerian hormone (AMH) level. Comparative analysis confirmed the superior comprehensive performance of the random forest model.

**Conclusion:**

This study successfully developed a robust machine learning model for predicting CHH treatment outcomes. It not only validates the methodological utility of ML in small-sample rare disease research but also elucidates the physiological basis of treatment response through interpretable feature clusters. The model provides clinicians with a quantifiable tool for risk stratification and paves the way for personalized therapeutic decision-making in CHH.

## 1 Introduction

Congenital Hypogonadotropic Hypogonadism (CHH) is a rare genetic disorder characterized by defects in the Hypothalamic-Pituitary-Gonadal (HPG) axis, with an incidence rate of approximately 1/10,000 to 1/84,000, showing significantly higher prevalence in males than in females (male-to-female ratio≈3–5:1) (Young et al., 2019). The core pathological mechanism involves impaired migration or dysfunction of Gonadotropin-Releasing Hormone (GnRH) neurons, resulting in insufficient gonadotropin secretion, which subsequently causes arrested gonadal development, absence of secondary sexual characteristics, and impaired fertility (Boehm et al., 2015). Currently, the mainstream clinical fertility induction therapy regimens include GnRH pulse therapy and combined hCG/hMG replacement therapy, which have been demonstrated to effectively initiate pubertal development and restore gonadal function in partial patients, achieving final spermatogenesis rates ranging from 64% to 95% across different studies (Young et al., 2019).

Existing research on factors influencing treatment outcomes in male CHH patients has primarily been confined to traditional statistical univariate or multivariate regression analyses. Reported associations include history of cryptorchidism, baseline testicular volume, baseline Inhibin B (INH-b) and anti-Müllerian hormone (AMH) levels, as well as the presence of positive genetic mutations (Rohayem et al., 2017; Cho et al., 2025; Pitteloud et al., 2002; Cangiano et al., 2021). However, such approaches exhibit significant limitations: firstly, they struggle to integrate multi-dimensional high-dimensional data encompassing clinical examinations, serological markers, and genetic data, often overlooking synergistic effects among indicators; secondly, they fail to provide a precise quantitative ranking of contributing factors’ impact magnitudes, making it difficult to meet individualized clinical management needs; thirdly, research subjects were predominantly adults or mixed adult-adolescent cohorts, with scarce studies focusing exclusively on adolescent male patients. The limited sample size constrains the application of statistical methodologies. In recent years, machine learning techniques have rapidly expanded within the medical domain. Their robust capabilities in non-linear fitting and high-dimensional feature mining have demonstrated significant advantages in areas such as diabetic complication prediction and tumor prognosis assessment (Gulshan et al., 2016; Esteva et al., 2017), offering a novel technical pathway for investigating treatment outcomes in rare diseases like CHH.

Artificial Intelligence (AI) has demonstrated translational potential in clinical medical research, with its core value manifested in the integration of high-dimensional heterogeneous data and enhancement of predictive capabilities. In the field of medical imaging, deep learning models can efficiently identify pathological lesions in X-rays and histopathological slides, enabling automated preliminary screening and quantitative analysis (Gulshan et al., 2016; Esteva et al., 2017; Hong et al., 2023; Esteva et al., 2019; Perez et al., 2019). Concurrently, machine learning can be leveraged to integrate multidimensional clinical data (medical records, genomics, vital signs) to construct disease risk prediction models, thereby facilitating early diagnosis and prognostic assessment (Cai et al., 2024; Yamamoto et al., 2022; Lee et al., 2023; Inoue, 2024; Lisik et al., 2025; AlShehhi et al., 2025; Guo et al., 2025). These technologies not only alleviate the burden of repetitive workloads for physicians but also serve as crucial auxiliary tools for enhancing diagnostic consistency and enabling personalized medicine by identifying patterns imperceptible to the human eye. However, in the field of rare diseases, particularly syndromes with exceptionally low incidence rates such as CHH, limited sample size and phenotypic heterogeneity constitute critical bottlenecks. Gonadal disorder follow-up is simultaneously impacted by privacy concerns, presenting considerable challenges in clinical data acquisition. In AI applications, limited by the number of complete follow-up cases, deep learning is highly susceptible to falling into overfitting, which consequently weakens the model’s extrapolation capability. In contrast, classical machine learning algorithms represented by random forest demonstrate superior bias-variance trade-offs in extremely small sample scenarios through their ‘shallow structure + ensemble strategy’: their splitting criterion is insensitive to feature scale and can naturally handle mixed-type variables; By introducing perturbations through bootstrap sampling and feature random subspace, they effectively reduce variance while preserving clinically interpretable pathways (Wang et al., 2022; Chen et al., 2023; Johnson et al., 2022). Therefore, in clinical research on rare diseases, prioritizing decision tree algorithms achieves dual objectives of robust prediction and knowledge discovery under data-constrained conditions (Banerjee et al., 2023). Currently, the application of ML learning in the CHH field remains at a nascent stage, and no relevant reports have been published. Building upon this, our study leveraged a single-center cohort with a relatively large sample size of CHH patients, integrating 15 baseline indicators across four categories to construct multiple ML predictive models. This approach aims to identify key influencing factors for Treatment outcomes while quantifying their weights, establish Critical Thresholds for core indicators, provide scientific evidence for individualized Therapeutic regimen development in CHH, and offer methodological references for precision medicine research in Rare Endocrine Diseases.

## 2 Materials and methods

### 2.1 Study participants

This single-center retrospective cohort study enrolled adolescent male patients with CHH who were diagnosed and received fertility induction therapy at our hospital’s endocrinology department between January 2010 and October 2025.

#### 2.1.1 Inclusion Criteria

Referencing our research group’s previously published article (Wang et al., 2017), the specific criteria were:

Advanced age group: 1. Male >14 years old without pubertal development, defined as testicular volume <4 mL; 2. Bone age ≥12 years 3. Baseline hormone levels indicating prepubertal status (serum androgen levels ≤20 ng/dL in males); 4. No elevation in baseline gonadotropin levels; 5. Absence of thyroid or growth hormone axis abnormalities; 6. Normal karyotype; 7. Developmental anomalies such as olfactory bulbs or olfactory tracts may be observed in the hypothalamic and pituitary regions, but no space-occupying lesions are present; 8. May be accompanied by micropenis, cryptorchidism, or hypospadias;

Younger age group: 1. Males >10 years old but ≤14 years old; 2. Meets criteria 3 to 8 of the advanced age group; 3. Positive family history of CHH; 4. According to ACMG standards, genetic testing reveals pathogenic or likely pathogenic mutations in candidate genes for CHH.

Patients without pubertal progression: 1. Testicular volume >4 mL, or basal testosterone level >20 ng/dL, or LH/FSH levels reaching early pubertal levels; 2. Presence of olfactory abnormalities or imaging-confirmed dysplasia of olfactory bulbs/tracts; 3. Adolescents demonstrating no pubertal progression during ≥6 months of clinical follow-up (Puberty Arrest) meeting definitive diagnostic criteria for Kallmann Syndrome were also enrolled.

#### 2.1.2 Exclusion criteria

① Identifiable etiologies (e.g., confirmed chromosomal abnormalities, trauma, surgery) or other known disorders causing hypogonadism (e.g., Prader-Willi Syndrome with sexual infantilism); ② Chronic systemic diseases (e.g., uremia, thalassemia, poorly controlled diabetes); ③ Protein-energy malnutrition; ④ Eating disorders (e.g., anorexia nervosa, hyperphagia); ⑤ Intracranial space-occupying lesions (e.g., pituitary tumors or post-operative status); ⑥ Absence of fertility-inducing treatment (GnRH pump or gonadotropin therapy); ⑦ Lost to follow-up within 6 months of treatment initiation.

#### 2.1.3 Treatment outcomes

1. Nocturnal emission group: Fertility induction therapy for at least 6 months, during which baseline Testosterone progressively reached >200 ng/dL, with testis and penis enlargement, culminating in nocturnal emission confirmed by patient or guardian report and documented by the follow-up physician during regular outpatient interviews (typically occurring within 12–24 months, though extended treatment duration was required in some cases).
2. Non-ejaculation group: Fertility induction therapy for at least 6 months, during which baseline testosterone remained <200 ng/dL with minimal testis/penis growth; or cases exhibiting initial testosterone elevation (>200 ng/dL) with genital development, but subsequently declined to <200 ng/dL with arrested/reversed genital growth, leading to therapy discontinuation and transition to testosterone replacement therapy.

Ultimately, 65 patients were included and categorized into the nocturnal emission group (55 cases) and non-ejaculation group (10 cases) based on treatment outcomes.

### 2.2 Data sources and indicator classification

#### 2.2.1 Data collection

Research data were sourced from the hospital’s electronic medical record system, laboratory information management system, and genetic testing database. Baseline patient information was independently extracted by two endocrinologists and cross-verified to ensure data accuracy. Penile length was measured in the flaccid state as the stretched length from the pubic bone to the tip of the glans, using a rigid ruler pressed firmly against the pubic symphysis to compress the suprapubic fat pad. Penile diameter was measured at the mid-shaft. All measurements were performed by experienced pediatric endocrinologists.

#### 2.2.2 Indicator system

A total of 15 pre-treatment baseline indicators across 4 major categories were collected as follows: ① Medical history: treatment initiation age, history of cryptorchidism, family history of constitutional delayed development or menstrual irregularities, disease type, presence of dual CHH (defined as testosterone levels <100 ng/dL following standard or extended human chorionic gonadotropin (HCG) stimulation testing), and treatment modality. ② Physical examination: testicular volume (measured using Prader orchidometer), penile diameter, penile length; ③ Serological indicators: luteinizing hormone (LH), FSH, testosterone, AMH, INH-b; ③ Genetics: genetic testing for phenotype-associated gene variants.

### 2.3 Therapeutic methods

Fertility induction therapy consisted of either gonadotropin-releasing hormone (GnRH) pulsatile pump therapy or combined gonadotropin therapy. The GnRH pulsatile pump regimen involved subcutaneous infusion of 5–12 µg per pulse every 90 min, delivering 16 pulses per 24 h. Initial dosing was conservative, with subsequent titration based on Serum Testosterone Levels, targeting a concentration of 200–500 ng/dL. Combined gonadotropin therapy employed two regimens: Protocol 1 involved initial monotherapy with hCG (1000–2000 IU) administered intramuscularly every other day or twice weekly. Upon achieving a testosterone level of 200 ng/dL, human menopausal gonadotropin (hMG) 75 IU was added weekly; protocol 2 utilized combined administration of hCG (500–2000 IU) and hMG (75–150 IU) administered 1–3 times weekly. Throughout treatment, hCG doses were adjusted to maintain Testosterone within target range.

All patients underwent follow-up at months 1 and 3 post-treatment initiation, subsequently every 3 months, encompassing hormone level assessments and physical examinations. Serum testosterone Levels were monitored 12–24 h post-hCG/HMG administration or during continuous GnRH pump therapy. Testicular volume was assessed using Prader orchidometer and ultrasonography, with bilateral mean values used for analysis.

### 2.4 Research instruments

All statistical analyses and machine learning modeling were conducted on MATLAB R2024b, implementing data cleansing, stratified cross-validation, Synthetic Minority Over-sampling Technique (SMOTE), Grid Search, and random forest training to ensure reproducible results.

### 2.5 Research methods

**FIGURE 1:**
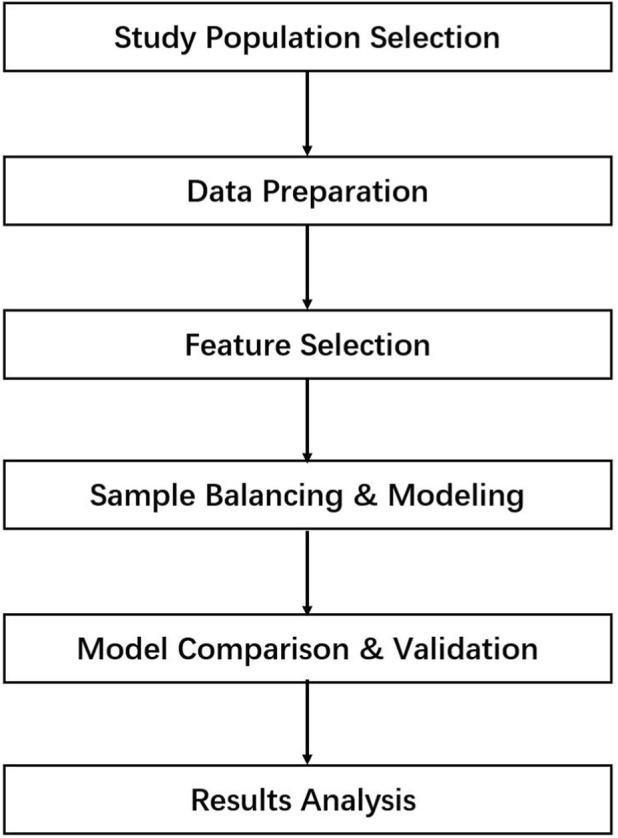
Flowchart of research methodology.

Figure 1 illustrates the research methodology workflow. This study employed a retrospective case-control design to develop and validate a machine learning algorithm-based predictive model for treatment outcomes in CHH. The research methodology was divided into six phases: Initially, Inclusion Criteria for study subjects were established, and subjects were retrieved from the electronic health record (EHR) system. Clinical data then underwent standardized preprocessing, including missing value imputation (median imputation for continuous variables, mode imputation for categorical variables) and low-variance feature filtering. Subsequently, multi-dimensional feature screening was conducted through univariate statistical analysis (Mann-Whitney U test for continuous variables, Chi-square test for categorical variables) combined with random forest feature importance ranking to identify key predictors. To address the Class Imbalance Problem, SMOTE was utilized to enhance the model’s recognition capability for the minority class. During the core modeling phase, the random forest algorithm was employed, and model performance was evaluated through 5-fold stratified cross-validation. Optimal prediction thresholds were determined through ROC curve analysis and Youden index calculation, with specific threshold recommendations provided for different clinical scenarios (avoiding missed diagnosis, avoiding overtreatment, and routine diagnosis). Through systematic comparison with control models including SVM, Logistic Regression, and k-NN, the predictive efficacy and clinical applicability of the developed model were comprehensively evaluated. Ultimately, the influencing factors of treatment outcomes were investigated through feature importance analysis based on machine learning.

### 2.6 Data preprocessing

1. Missing value handling: Continuous indicators (e.g., LH, FSH levels) were imputed using the median of their respective groups (success/failure groups); Categorical indicators (e.g., genetic mutation status) were imputed using the mode. Ultimately, the proportion of missing data in all cases was <5%.
2. Outlier identification and handling: Outliers were identified using the boxplot method (IQR±1.5). Following clinical expertise assessment, non-clinically significant outliers were truncated, while clinically specific outliers were retained and annotated.
3. Low-variance Feature Filtering: Core function is to eliminate features with minimal numerical fluctuation and no outcome discriminability (e.g., homogeneous test indicators). By setting a 0.01 variance threshold, high-information features were screened while guaranteeing retention of minimum indicators. This approach reduced computational load, mitigated overfitting, and allowed subsequent modeling to focus on clinically discriminative indicators, thereby establishing the foundation for the precision and generalizability of the CHH treatment outcome prediction model.
4. Class Imbalance Correction: Due to differences in sample sizes between groups (success group: 55 cases, failure group: 14 cases), SMOTE was employed for oversampling to balance the training set sample distribution; Simultaneously, class weights were set during model training to mitigate the impact of sample imbalance on the model.
5. Data Standardization and Encoding: Continuous indicators were standardized using Z-score normalization; binary indicators (e.g., presence of genetic mutations) were encoded using 0–1 binary encoding.

### 2.7 Feature selection

The core objective of the feature selection step is to identify core features strongly associated with treatment outcomes (success/failure) and possessing high predictive value from preprocessed high-variance features. This serves to reduce model computational complexity, prevent overfitting, and enhance the clinical interpretability of the model. This step employs a dual logic of ‘preliminary screening via statistical tests + rigorous screening based on model importance,’ dynamically adapted to the characteristics of the clinical data. The specific workflow and its significance are outlined below:

First, univariate statistical tests were conducted for preliminary screening. Considering the differences between continuous (e.g., hormone levels) and categorical (e.g., therapeutic regimen) features in clinical data, appropriate methods were employed: Mann-Whitney U test (non-parametric test, given the non-normal distribution characteristics of clinical data) for continuous features and Chi-square test (to analyze feature-outcome associations) for categorical features. The P-values indicating associations between each feature and the outcome were calculated, with features demonstrating P < 0.05 selected as significant during the initial screening. Features lacking discriminative power (e.g., single-value features) were directly marked as having no value (P = 1) to ensure the statistical validity of the preliminary screening results.

Secondly, adaptive dimensional optimization was implemented by setting the maximum feature count. Considering the typically limited sample size in clinical research, the maximum feature count was dynamically adjusted based on sample size constraints: ≤50 samples used 1/5 features, ≤100 samples used 1/4 features, otherwise 1/3 features were utilized. Concurrently, upper and lower thresholds (2–20 features) were constrained to prevent overfitting caused by ‘high-dimensional data with limited samples,’ thereby balancing feature dimensions with model generalization capability to accommodate the scarcity characteristic of CHH clinical data.

Finally, feature importance-based rigorous screening was performed using the random forest algorithm. Temporary random forest models were trained to quantify feature importance through ‘Out-of-Bag Error Change,’ with the top 80% of high-importance features being selected; If fewer than 2 features remain post-screening, the fallback mechanism retains the optimal feature from initial screening to ensure the feature set meets minimum validity thresholds. The final output specifies both the number of retained features and their identities, explicitly identifying core predictive targets for CHH treatment outcomes. Through the above comprehensive workflow, 7 core features were ultimately selected from the original 15 baseline indicators for training the final random forest prediction model. This process corresponds to the ‘Feature Screening’ stage in Figure 1.

This comprehensive workflow balances statistical significance with predictive value by excluding irrelevant features and focusing on core clinical indicators (e.g., key hormone levels, treatment phase parameters). This approach enhances subsequent model training efficiency and generalizability while providing clinically actionable targets for decision-making, thereby strengthening the model’s clinical utility.

### 2.8 Development and training of machine learning models

#### 2.8.1 Model selection

Random forest model was selected as the primary methodology for this investigation. The random forest algorithm, as an ensemble tree-based model, accommodates nonlinear relationships, adapts to small-sample datasets, and outputs Feature Importance metrics, thereby aligning with the core requirements of this study. Through dual perturbation mechanisms—bootstrapping aggregation and feature random subspace—the random forest architecture inherently suppresses overfitting in our limited cohort of 65 CHH patients; Its robust handling of mixed data types, missing values, and nonlinear associations obviates stringent distributional assumptions and complex preprocessing. The ensemble voting mechanism seamlessly generates class probabilities, facilitating subsequent optimization of clinical decision thresholds; The built-in Out-of-Bag (OOB) error and Feature Importance metrics provide directly interpretable evidence for dimensionality reduction under lenient significance thresholds, balancing predictive performance with mechanistic insights.

#### 2.8.2 Stratified cross-validation design

In CHH clinical treatment outcome prediction studies, the core design of stratified k-fold cross-validation (k = 5) addresses both clinical data characteristics and the rigor requirements of clinical research.

1. Resolving the class imbalance problem in clinical data to ensure unbiased evaluation. In CHH clinical practice, ‘treatment failure’ samples are typically significantly fewer than ‘success’ samples. Conventional cross-validation may suffer from imbalanced sample distribution in individual folds (e.g., absence of failure samples in a particular fold), causing models to exhibit bias toward predicting the majority class. The stratified design ensures consistent class distribution across all folds by performing stratified sampling based on the ‘success/failure’ ratio of the original data. This approach enables the model to learn features of both outcome categories in each fold, preventing distorted evaluation due to sampling bias. Crucially, it guarantees objective assessment of predictive capability for clinically critical ‘failure outcomes’.
2. Maximizes the utilization of limited clinical samples to enhance evaluation reliability. Given the difficulty in obtaining clinical research samples and their limited quantity, stratified cross-validation employs an iterative ‘training-testing’ cycle design. This allows every sample to participate in both training and testing phases, maximizing information extraction from the dataset. Compared to single train-test splits, this approach significantly reduces evaluation error caused by random partitioning. The selection of 5-fold cross-validation balances evaluation efficiency and result stability, mitigating the risk of high variance from insufficient folds while avoiding computational redundancy from excessive folds. This approach ensures that core metrics (mean ± SD), including AUC and F1-score, more accurately reflect the model’s true generalization capability.
3. This design aligns with the stringent reliability requirements of clinical decision-making, thereby enhancing model trustworthiness. Clinical outcome prediction directly informs personalized treatment regimen development, making model stability of paramount importance. Stratified cross-validation demonstrates model performance consistency across distinct sample subsets by revealing metric fluctuations (e.g., AUC standard deviation) throughout multiple validation folds. Minimal fluctuations in metrics across folds indicate that the model maintains stable predictive capabilities for CHH patients with varying feature distributions, providing crucial reliability evidence for clinical implementation; this prevents clinical decision-making risks arising from model failure in specific patient subgroups. Furthermore, it reinforces the rigor of research conclusions and meets requirements for both academic validation and clinical translation. Stratified cross-validation represents the standardized evaluation methodology in clinical machine learning research, offering strong reproducibility that effectively verifies the model’s generalization capability is not attributable to chance sample partitioning. The multidimensional indicators (Accuracy, Precision, Recall, AUC) and their fluctuation ranges obtained through this study design provide robust quantitative support for the conclusion that ‘the model can effectively predict CHH treatment outcomes,’ enhancing the academic persuasiveness of the research findings and establishing a methodological foundation for subsequent clinical translation of the model.

#### 2.8.3 SMOTE oversampling

In this study, addressing the extremely imbalanced scenario with only 65 CHH cases and 10 positive events, SMOTE was employed to infuse ‘synthetic yet credible’ minority class samples into the random forest through feature-space interpolation, fundamentally mitigating model bias induced by class imbalance. The algorithm generates new points along the difference vectors between randomly selected minority class samples and their k-NN, preserving the original feature distribution while avoiding overfitting caused by simple duplication. Continuous variables and discrete variables were processed using linear interpolation and mode voting respectively, ensuring logical consistency across blood test results, physical examination indicators, and genetic history. After SMOTE balancing, the success-to-failure ratio in the training set was adjusted from 4.9:1 to approximately 1:1. This enabled random forest’s bootstrap sampling to be adequately exposed to failure samples during node splitting in each decision tree, thereby correctly learning the decision boundary and significantly improving Recall and F1-score. Meanwhile, the introduction of synthetic samples increases the coverage density in the feature space and reduces the sensitivity of decision tree partitioning to noise. Combined with random forest’s out-of-bag error estimation, this approach could further suppress the risk of overfitting. Notably, SMOTE operates exclusively on the training set, while the validation and test sets retain their original distributions. Consequently, performance metrics can still authentically reflect the model’s generalization capability in real clinical scenarios. For rare diseases such as CHH with extremely high data acquisition costs, SMOTE employs a low-cost ‘data augmentation’ strategy. This enables random forest to achieve statistical power commensurate with the sample size without increasing patient burden, thereby providing a robust foundation for subsequent feature screening, threshold optimization, and clinical interpretation.

#### 2.8.4 Hyperparameter tuning

This code implementation does not include explicit hyperparameter optimization processes (such as grid search, Bayesian Optimization, or other iterative optimization methods). Instead, it employs a core strategy of ‘self-adaptive parameter adjustment + empirical value setting’ to accommodate the characteristics of CHH clinical data. Firstly, key hyperparameters (e.g., the number of trees in random forest, cross-validation folds, SMOTE neighbor count) were predetermined with empirical values through a structured parameter set, without comparative screening of multiple parameter combinations. Secondly, model parameters are dynamically adjusted according to data scale: in random forest training, the ‘number of features per tree’ (max_features) is adaptively determined as the ‘square root of feature count’, while the ‘minimum leaf node size’ (min_samples_leaf) is adjusted based on total sample size (not less than 2 and set to 1/20th of the sample count). During the feature selection phase, the maximum feature count was dynamically determined based on sample size (≤50 samples: 1/5 features, ≤100 samples: 1/4 features, otherwise: 1/3 features) to balance dimensions and generalizability. Although not explicitly optimized, this design accommodates the limited availability of clinical samples. By integrating empirical values with data adaptability, it maintains model stability while simplifying computational workflows, effectively mitigating overfitting risks associated with hyperparameter tuning in small sample sizes.

### 2.9 Feature importance evaluation methodology

The random forest model developed in this study, constructed through a comprehensive workflow of ‘data preprocessing-feature screening-class balancing-stratified validation’, demonstrated excellent and stable predictive performance, indicating substantial potential for clinical application. The core strength of the model lies in its optimal adaptation to clinical data characteristics. By adopting an implicit hyperparameter tuning strategy, it streamlines the workflow while ensuring reliability, with specific manifestations and values as follows:

Feature importance assessment within our random forest model serves as a pivotal step for screening core predictive indicators of CHH treatment outcomes and enhancing model interpretability. Centered on ‘OOB Permuted Predictor Delta Error’ to quantify feature value, this approach permeates the entire workflow from feature selection to result visualization, aligning with both clinical data characteristics and modeling requirements.

Regarding evaluation principles and implementation logic, the model validates Feature Importance through ‘OOB’ data: For each feature, its values in OOB data are randomly permuted, followed by recalculation of the model’s out-of-bag prediction error; The feature importance value is calculated as the difference between post-permutation error and original error. A larger difference indicates a greater contribution of the feature to model prediction, and its absence would cause significant degradation in model performance. This method requires no additional validation set, adapting to the limited availability of clinical samples, while effectively circumventing interference from overfitting in evaluation results.

The application in the modeling workflow comprises two core phases: First, rigorous screening during feature selection. Based on preprocessed high-variance features, we train a provisional random forest comprising 50 decision trees to compute feature importance. Features are ranked in descending order of importance, with the top 80% high-importance features selected. A safeguard mechanism ensures retention of at least two features. This approach compensates for the limitation of univariate statistical tests that focus solely on pairwise ‘feature-outcome’ associations, further eliminating redundant features from the perspective of model prediction logic. Secondly, the visualization of the final model involved training the definitive random forest on the complete balanced dataset (post-SMOTE). Feature Importance values were extracted and presented in a bar chart, clearly annotating the predictive weights of clinical characteristics (e.g., hormone levels, treatment phase indicators) to provide an intuitive basis for clinical interpretation.

The core value of this evaluation method lies in striking a balance between modeling performance and clinical significance: on one hand, by screening high-importance features, it reduces model computational complexity and enhances predictive stability, avoiding overfitting caused by irrelevant features; On the other hand, the quantified Feature Importance results delineate core therapeutic targets for predicting CHH treatment outcomes, transforming the model from a ‘black box’ into an interpretable clinical tool. This empowers clinicians to identify key indicators influencing therapeutic efficacy, provides data-driven support for formulating personalized treatment regimens, and enhances the clinical translational value of research findings.

### 2.10 Model performance evaluation metrics

This study employs multi-dimensional metrics to comprehensively evaluate the random forest model’s performance in predicting clinical treatment outcomes for CHH. Core metrics include Accuracy, Precision, Recall, F1 score, and AUC value, specifically adapted to address class imbalance characteristics of clinical data to ensure objective and practical evaluation. Accuracy reflects the model’s overall prediction correctness, serving as a fundamental evaluation benchmark; precision measures the proportion of true positives among samples predicted as ‘failure to achieve nocturnal emission post-treatment,’ preventing excessive misjudgment of unsuccessful cases. Recall focuses on the detection capability for genuinely unsuccessful cases, aligning with the clinical core need for early warning of adverse outcomes. The F1 score is the harmonic mean of precision and recall, balancing the trade-off between these two metrics and adapting well to the evaluation of imbalanced datasets. The AUC value, calculated via the ROC curve, comprehensively reflects the model’s ability to distinguish between ‘treatment success’ and ‘treatment failure,’ offering enhanced stability. All metrics were calculated based on a 5-fold stratified cross-validation, reporting the mean ± standard deviation. This approach mitigates the randomness of single evaluations, quantifies model variability, and provides robust quantitative support for the reliability of the model’s clinical application.

## 3 Results

**FIGURE 2:**
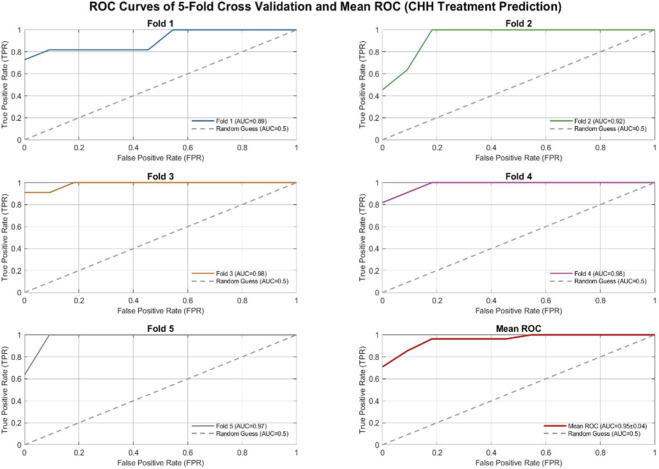
Random Forest model 5-fold stratified cross-validation.

As illustrated in Figure 2, the model demonstrates exceptional comprehensive performance and robust stability in training outcomes. Core evaluation metrics based on 5-fold stratified cross-validation exhibited outstanding performance: Accuracy reached 0.84 ± 0.12, indicating a high level of overall predictive correctness; precision was 0.82 ± 0.13, signifying a high proportion of true positives among cases predicted as ‘failure to achieve nocturnal emission post-treatment’, which can effectively mitigate clinical decision-making risks associated with excessive misjudgment of treatment failures; recall of 0.89 ± 0.08 reflects the model’s strong detection capability for genuine failure samples, aligning with the clinical imperative for early warning of adverse outcomes; F1 score 0.85 ± 0.10 indicates balanced coordination between Precision and Recall, demonstrating adaptability to the class imbalance characteristic in clinical data; the particularly critical AUC value reached 0.95 ± 0.04, signifying the model’s exceptional comprehensive ability to differentiate between ‘successful nocturnal emission post-treatment’ and ‘failure to achieve nocturnal emission post-treatment’ outcomes, with minimal standard deviation reflecting high consistency in model performance across different sample subsets.

The stability of model performance benefits from scientific workflow design: balancing Minority class (failure samples) distribution via SMOTE oversampling prevents model bias toward Majority class; stratified cross-validation ensures consistent sample distribution across all folds with the overall dataset, enhancing evaluation reliability; during the feature selection phase, core clinical indicators were screened by combining statistical testing with random forest importance assessment, simultaneously simplifying model architecture and enhancing interpretability. In summary, this model not only demonstrates predictive advantages of high accuracy and strong discriminative capability but also reduces the barrier to application by adapting to clinical data characteristics. It empowers clinicians to precisely identify key factors influencing treatment outcomes and provides reliable data support for formulating personalized treatment regimens for CHH patients.

### 3.1 Quantitative ranking of influencing factors for treatment outcomes

#### 3.1.1 Inherent feature importance results of the model

**FIGURE 3:**
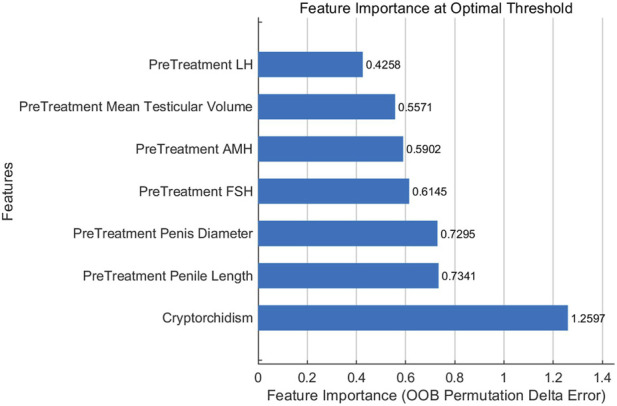
Model feature importance ranking.

As shown in Figure 3, the feature importance ranking of the random forest model is as follows: Cryptorchidism (Out-of-Bag Error Change 1.2597), Pre-Treatment Penile Length (0.7341), Pre-Treatment Penis Diameter (0.7295), Pre-Treatment FSH (0.6145), Pre-Treatment AMH (0.5902), Pre-Treatment Mean Testicular Volume (0.5571), Pre-Treatment LH (0.4258). Notably, the feature importance value of ‘history of cryptorchidism’ (1.2597) as a binary variable is significantly higher than that of other continuous variables, highlighting its core position in the predictive model.

#### 3.1.2 Determination of critical indicator thresholds

This study developed a CHH treatment outcomes prediction model through ROC curve analysis, identifying the optimal critical threshold and core predictive features to provide a quantitative basis for formulating personalized clinical treatment strategies. The results are analyzed as follows.

**FIGURE 4:**
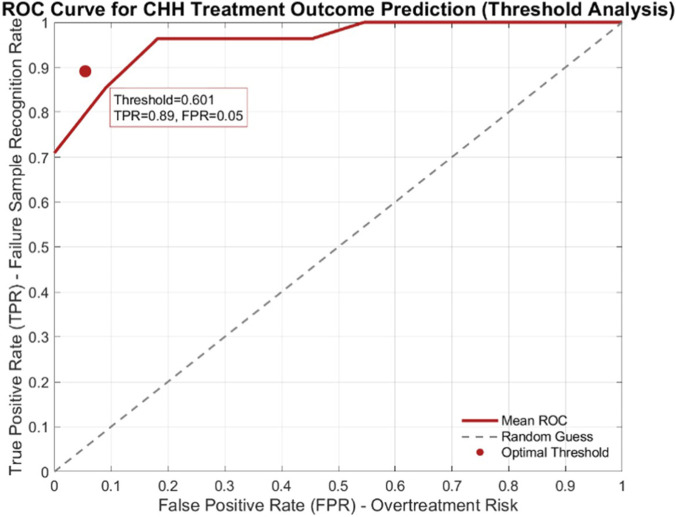
Roc curve of the Model’s optimal threshold.

Critical threshold analysis revealed (Figure 4) that based on the principle of maximizing the Youden index, the optimal threshold across model folds was 0.601 ± 0.137. This threshold achieved a treatment failure identification rate (TPR) of 0.89 with only a 0.05 misclassification rate (FPR) for successfully treated nocturnal emission cases, yielding a Youden index of 0.836. These results demonstrate the model’s outstanding predictive accuracy and clinical utility. To address distinct clinical management needs, as Table 1 shows, this study proposes a stratified threshold recommendation strategy: Scenario 1 (prioritizing avoidance of false negatives in treatment-nonresponsive patients) adopts a 0.501 threshold, ensuring an 87% failure sample identification rate at the cost of a marginally elevated false positive rate (0.10), applicable for high-risk population screening; Scenario 2 (prioritizing avoidance of overtreatment) employs a 0.701 threshold, balancing medical resource wastage while maintaining an 84% failure sample identification rate, suitable for conservative management of low-risk patients; Scenario 3 (standard clinical management) utilizes the optimal 0.601 threshold, achieving precise equilibrium between risk of false negatives and overtreatment.

**TABLE 1:** Recommended clinical thresholds for CHH treatment prediction.

Scenario	Threshold	True positive rate (TPR)	False positive rate (FPR)
Prioritizing avoidance of false negatives in treatment-nonresponsive patients	0.501	0.87	0.16
Prioritizing avoidance of overtreatment	0.701	0.86	0.22
Standard clinical management	0.601 (Youden optimal threshold)	0.89	0.05

The stratified cutoff thresholds established in this study can be tailored to different clinical scenario needs. Cryptorchidism, pretreatment external genitalia development, and hormone-related indicators (including AMH, INH-b, and FSH) serve as core predictive factors for CHH treatment outcomes. Their synergistic application provides critical references for treatment risk assessment and personalized regimen optimization in CHH patients.

### 3.2 Comparison with other machine learning methods

**FIGURE 5:**
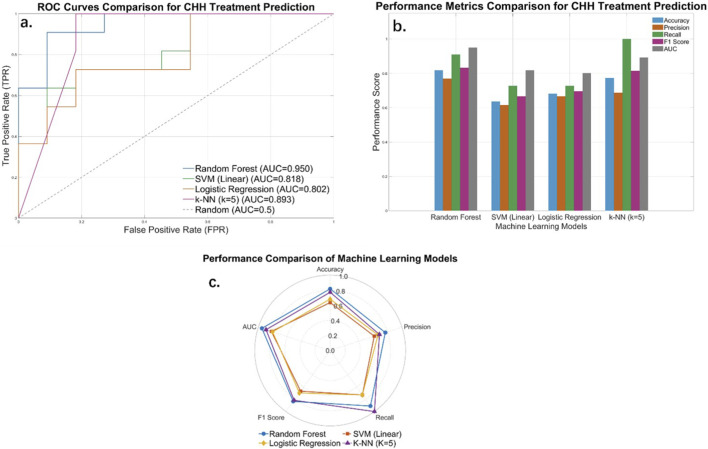
Performance comparison of different machine learning methods: **(a)** Comparative ROC Curves of Models. **(b)** Bar chart of Model Performance Differences. **(c)** Radar Chart of Model Performance Disparities.

This study conducted comparisons with three classical machine learning models—SVM, Logistic Regression, and k-NN—by replacing the random forest model in the research procedure. As illustrated in Figure 5a, the model comparison results demonstrate that the random forest model (AUC = 0.9504) achieved optimal performance in predicting CHH treatment outcomes, with its AUC value being significantly higher than those of other models (an 11.29% improvement), showcasing robust discriminatory capacity. The ROC curve comparison graph visually demonstrates the classification performance of each model: The random forest curve is closest to the top-left corner, followed by k-NN, while the SVM and Logistic Regression curves are nearer to the diagonal, indicating limited discriminative capability. Notably, while k-NN slightly underperforms random forest in AUC (0.8926 vs. 0.9504), its Recall reaches 1.0, achieving complete identification of cases without nocturnal emission. This characteristic positions it as an effective supplementary tool for high-risk screening.

The bar chart in Figure 5b reveals differences in model performance across multiple dimensions. Random forest demonstrated superior comprehensive performance and computational efficiency, leading in Accuracy (0.8182), F1 score (0.8333), and AUC metrics. While achieving exceptional Recall (1.0), k-NN’s performance came at the cost of slightly lower Precision, yet provides significant value for clinical early-warning systems. SVM and Logistic Regression showed k-NN balanced but overall lower performance across all metrics, indicating the limitations of Linear Models when handling this non-linear clinical data.

Figure 5c radar chart geometrically visualizes model characteristics: random forest displays a near-pentagonal balanced profile, demonstrating robustness across all evaluation dimensions; The curve demonstrated significant convexity along the recall axis, exhibiting an asymmetric profile indicative of high sensitivity yet relatively insufficient specificity; the compact contours of SVM and Logistic Regression confirmed their limited performance. Comprehensive visualization analyses revealed that random forest exhibited optimal balance, discriminative power, and practicality for CHH treatment prediction, establishing it as the preferred model for clinical decision support. Meanwhile, k-NN’s high-recall characteristic renders it suitable for auxiliary screening of high-risk cases.

## 4 Discussion

This study successfully achieved high-performance prediction of fertility induction therapy outcomes (marked by nocturnal emission) in adolescent male patients with CHH by constructing a predictive model based on the random forest algorithm. The model demonstrated exceptional discriminative capability (AUC: 0.95 ± 0.04) and stability, which not only validates the methodological advantages of machine learning in rare disease and small-sample clinical research, but also provides novel insights into the pathophysiological heterogeneity of CHH and the underlying mechanisms of variability in treatment response through its identification and quantified weight assignments of core predictive features.

### 4.1 Methodological innovation

Compared with traditional studies relying on univariate or multivariate regression analysis, the random forest algorithm employed in this study demonstrates fundamental advantages (Banerjee et al., 2023). CHH treatment outcomes are influenced by a complex interplay of multidimensional, nonlinear factors including genetic background, baseline developmental status, hormone levels, and therapeutic response. Previous research on predicting CHH treatment outcomes predominantly relied on linear regression or univariate analysis. For instance, studies by Miyagawa et al. (2005) and Liu et al. (2016) established baseline testicular volume and history of cryptorchidism as significant prognostic indicators through retrospective analysis; The prospective study by Rohayem et al. (2017) further corroborated that larger baseline testicular volume, higher baseline INH-b, and elevated AMH levels are associated with superior treatment outcomes. Although these studies uncovered valuable clinical markers, their methodological approaches struggled to account for complex interactions among variables. Research by Sykiotis et al. (2010) revealed that some CHH patients present with comorbid pituitary gland or testis defects, highlighting the complexity of the disease mechanism. Traditional statistical methods exhibit poor compatibility with high-dimensional data, fail to capture intricate feature interactions, demonstrate susceptibility to subjective assumptions during feature screening, and offer limited predictive accuracy. As an ensemble learning algorithm, random forest robustly handles complex inter-variable relationships and quantifies feature importance through ranking by constructing numerous decision trees and aggregating their outcomes (Wang et al., 2022; Chen et al., 2023; Johnson et al., 2022; Banerjee et al., 2023). This study revealed that the random forest model’s overall performance significantly outperformed linear or simple non-linear models such as Logistic Regression and SVMs. This confirms that in rare disease research like CHH with complex pathophysiological mechanisms, employing machine learning models capable of parsing high-dimensional interactions is crucial for enhancing predictive accuracy and clinical decision-making capabilities. This offers a reliable and interpretable methodological paradigm for precision medicine research in rare endocrine diseases.

#### 4.1.1 Comparison with other algorithms

The random forest model possesses innate advantages in handling the complexity of clinical data. CHH treatment outcomes are influenced by multifactorial interactions, including nonlinear relationships among patient baseline characteristics, therapeutic regimen, and laboratory indicators. By integrating multiple decision trees, random forest can effectively capture these complex interaction effects and threshold effects, whereas linear models (such as SVM and Logistic Regression) demonstrate limited capability in modeling such nonlinear relationships. Concurrently, random forest demonstrates insensitivity to feature scale, circumventing information loss inherent in standardization—a characteristic to which k-NN exhibits high dependency. Furthermore, the feature importance ranking capability of random forest provides crucial support for analyzing contributing factors to treatment outcomes.

Model comparison results further reveal distinct clinical application scenarios across algorithms. Random forest achieved optimal comprehensive performance (AUC, F_1_ score), establishing its suitability as a benchmark model for routine clinical decision support. Notably, the k-NN model attained a Recall of 1.0, indicating exceptional recognition sensitivity for cases involving failure to achieve nocturnal emission post-treatment. Although its precision is relatively low, the k-NN model can serve as an effective complementary tool in clinical scenarios focused on screening and avoiding missed diagnosis of patients with poor treatment responses. This suggests that future clinical decision support systems could consider integrating multiple models to enable flexible invocation based on different clinical priorities (e.g., high sensitivity vs. high specificity).

### 4.2 In-depth analysis of core predictive factors

The top five core predictive features identified by this model—cryptorchidism, pretreatment penile length, penile diameter, FSH levels, and AMH levels—are not isolated indicators but collectively reflect the severity of HPG axis defects, baseline gonadal status, and accumulated ‘micro-stimulation’ by endogenous gonadotropins. Their physiological significance requires thorough interpretation within the context of individual growth and sexual maturation processes.

#### 4.2.1 Cryptorchidism

In this study, cryptorchidism emerged as the strongest negative predictor of treatment outcomes with the highest feature importance (Out-of-Bag Error Change: 1.2597). This data finding is highly consistent with long-standing clinical understanding: in clinical practice, a history of cryptorchidism, especially bilateral cryptorchidism, is often associated more severe functional impairment of the hypothalamic-pituitary-gonadal axis and may predict a less favorable response to fertility induction therapy.

During normal male embryonic development, testicular descent relies on Insulin-like factor 3 (INSL3) and testosterone produced by fetal Leydig cells. The key mechanism underlying cryptorchidism in CHH patients stems from deficient GnRH secretion during the fetal period, resulting in gonadotropin (LH/FSH) deficiency which consequently impairs testosterone and INSL3 synthesis. Therefore, a history of cryptorchidism, particularly bilateral cryptorchidism, strongly suggests severe functional defects in the HPG axis during the fetal period. This indicates potentially compromised developmental foundations of testicular Leydig cells and Sertoli cells. This congenital impairment significantly reduces the testicular potential for proliferative and differential responses to exogenous gonadotropin stimulation (GnRH or hCG/hMG) during puberty, consequently hindering the initiation of spermatogenic function. In this study, the prevalence of cryptorchidism was significantly lower in the nocturnal emission group than in the non-ejaculation group, quantitatively demonstrating this risk association. Raivio et al. (2007) found that patients lacking postnatal hypothalamic-pituitary-testicular axis activation (often accompanied by cryptorchidism) exhibited significantly reduced responsiveness to recombinant human follicle-stimulating hormone (r-hFSH). Studies by Liu et al. (2016) and Cangiano et al. (2021) consistently identified a history of cryptorchidism as a predictor of suboptimal therapeutic response. This collectively indicates the profound and lasting impact of gonadal dysgenesis during the fetal period.

#### 4.2.2 Pre-treatment penile length and diameter

The growth and development of the penis serves as a classic indicator of androgen action (primarily dihydrotestosterone). In normal males, penile size growth is extremely slow prior to pubertal onset. Due to lack of pubertal onset, CHH patients’ penises typically remain in a prepubertal state. However, some infants may experience a transient and incomplete ‘minipuberty’ during infancy, during which pulsatile secretion of endogenous gonadotropins can lead to partial androgen production, thereby promoting initial penile growth. Therefore, relatively larger pretreatment penile size may suggest that the patient experienced a more active minipuberty during infancy, suggesting residual hypothalamic-pituitary function or potential responsiveness to GnRH stimulation. Specifically, greater penile size serves as evidence of prior activation of the endogenous HPG axis, indicating that the gonadal target organ (corpus cavernosum of the penis) has previously responded to androgen stimulation. This indirectly suggests that testicular Leydig cells may possess enhanced functional reserve capacity, potentially leading to superior responsiveness to exogenous therapeutic intervention.

#### 4.2.3 Pre-treatment FSH and AMH

FSH and AMH constitute core indicators for evaluating Sertoli cell function. Their predictive value reveals the baseline status of the seminiferous tubule microenvironment.

FSH: In normal physiology, FSH is secreted by the pituitary gland and acts directly on Sertoli cells, promoting their proliferation and synthesis of various nutrients to provide a microenvironment for spermatogenesis. In CHH patients, FSH levels are typically in the low range. This study found that although all were within the low range, patients with higher pre-treatment FSH levels had higher treatment success rates. This may imply that even in a state of overall hypogonadotropism, those who maintain relatively higher baseline FSH levels may possess better gonadotroph function for FSH secretion in the pituitary gland or retain some responsiveness to very low-frequency GnRH pulses that may exist in the hypothalamus. Higher baseline FSH levels may provide weak yet persistent trophic support to Sertoli cells, enabling faster initiation and maintenance of spermatogenesis upon subsequent intensive gonadotropin therapy.

AMH: Continuously secreted by immature Sertoli cells, serves as a specific biomarker for male fetal sexual differentiation and prepubertal Sertoli cell function. Following pubertal onset, AMH secretion dramatically declines as Sertoli cells mature under FSH and testosterone stimulation to support spermatogenesis. In CHH patients, AMH levels typically remain at prepubertal high levels due to failure of puberty to initiate. In this study, higher pretreatment AMH levels served as a positive predictor of treatment success. This can be interpreted as: Elevated AMH levels reflect a larger population or more immature state of Sertoli cells. A larger Sertoli cell reserve implies that more cells can be activated, mature, and undertake spermatogenesis-supporting functions upon exogenous FSH stimulation, thereby providing a more robust foundation for successful spermatogenesis.

#### 4.2.4 Other relevant factors: Pretreatment testicular volume and LH

Although not ranked among the top five factors, pretreatment testicular volume and LH also hold significant physiological implications. Testicular volume is predominantly constituted by seminiferous tubules (containing Sertoli cells and germ cells), with its size directly reflecting the spermatogenic epithelial capacity. Larger pretreatment testicular volume typically indicates better developmental foundation of seminiferous tubules. LH primarily stimulates Leydig cells to produce testosterone. In CHH patients, basal LH levels are markedly low; however, subtle variations may still reflect minor differences in pituitary LH reserves or sensitivity to GnRH. Similar to FSH, LH serves as a window for assessing residual pituitary function.

In summary, the feature clusters identified by the model systematically evaluate the baseline status of CHH patients from different yet complementary dimensions: cryptorchidism reflects gonadal dysgenesis resulting from severe functional deficiency of the HPG axis during the fetal period; pre-treatment penile size can serve as an indirect indicator of endogenous androgen exposure levels during the minipuberty phase of infancy, demonstrating the historical responsiveness of target organs; basal FSH and AMH levels collectively assess the functional status and reserve capacity of Sertoli cells; pre-treatment testicular volume manifests as a comprehensive morphological indicator of the aforementioned developmental process. These parameters collectively establish a continuous assessment system spanning from the fetal period through infancy to puberty, enabling integrated evaluation of the severity spectrum of HPG axis dysfunction and the developmental foundation of gonadal function in patients. Consequently, patients presenting with a constellation of features including a history of cryptorchidism, micropenis, small testis, low FSH, and low AMH levels typically indicate more severe and comprehensive HPG axis functional impairment, carrying an elevated risk of lower therapeutic response. Conversely, if these indicators are relatively closer to the normal range, it often suggests partial residual function of the hypothalamic-pituitary-gonadal axis, indicating a greater likelihood of achieving sufficient response to gonadotropin therapy.

Furthermore, some indicators (e.g., specific genetic variants, treatment regimens) did not emerge as core predictors, as their effects may have been subsumed by more direct phenotypic predictors. This suggests that for predicting early treatment response in CHH, a patient’s baseline anatomical and endocrine functional status may hold more direct indicative value than specific genetic background or the initial treatment choice. Future work integrating multi-omics data and refined genetic analyses is needed to elucidate their role.

### 4.3 Clinical translation value and limitations

This CHH treatment outcome prediction model demonstrates significant clinical utility. First, the top five core features comprehensively characterize baseline status across multiple dimensions including reproductive development, hormone levels, and physical development, enabling precise assessment of disease severity and therapeutic response potential while providing quantitative foundations for risk stratification. Second, stratified cutoff thresholds accommodate varying diagnostic and therapeutic needs, ensuring minimized risk of false negatives in high-risk patients while preventing overtreatment in low-risk patients, thereby balancing therapeutic safety and cost-effectiveness. Third, by identifying critical warning signals and establishing personalized thresholds, the model assists clinicians in developing precise intervention strategies, enhancing treatment success rates while optimizing healthcare resource allocation, providing reliable technical support for personalized CHH management.

This study has several limitations. First, as a single-center retrospective study with a limited sample size, although robustness was enhanced through methods such as SMOTE and cross-validation, the conclusions still require external validation in prospective, multicenter, larger-scale cohorts. In particular, the model’s performance may vary across different populations, treatment initiation criteria, and different gonadotropin preparations or administration regimens. The excellent performance based on internal validation (AUC: 0.95 ± 0.04) suggests the model’s potential value, but its universal applicability in broader clinical settings must be strictly validated through future collaborative research, for instance, by employing methods like federated learning (Süwer et al., 2026), on independent patient cohorts. This is an indispensable next step for translating the research findings into a clinical decision support tool. Second, although the baseline characteristics included in this study encompass commonly used clinical indicators, future research could integrate data from additional dimensions such as imaging features, more comprehensive genetic profiles, or dynamic hormonal changes during continuous treatment to further enhance the predictive accuracy of the model. Finally, due to ethical considerations, developmental stage, and patient compliance, routine semen analysis in adolescent CHH patients is significantly limited. Therefore, we chose “nocturnal emission” as the predictive endpoint of this model. While it serves as an important intermediate milestone for the initiations of pubertal development and spermatogenesis, long-term fertility outcomes (e.g., semen parameters, pregnancy rates) require confirmation through longer-term follow-up. We fully acknowledge that “nocturnal emission” is a surrogate marker, not a direct measure of fertility. However, at the initiation of fertility induction therapy, which may span several years, its clinical relevance lies in providing near-term decision support: it enables the early prediction of “non-responders” who are unlikely to achieve this initial physiological milestone. This allows clinicians to consider timely adjustments to the treatment plan (e.g., drug dosage or type), potentially sparing patients from prolonged, ineffective treatments that carry psychological and economic burdens. Consequently, the primary purpose of this model is to predict the initiation of treatment response, providing a quantitative basis for early risk stratification, rather than directly predicting ultimate fertility potential. Establishing the association between this early surrogate endpoint and long-term reproductive outcomes is a key objective of our subsequent long-term follow-up studies.

### 4.4 Potential perspective on endocrine-immune interplay

This study focuses on the association between intrinsic HPG axis defects and treatment response in CHH. However, accumulating evidence suggests a bidirectional relationship between hypogonadism and immune regulation. Sex hormones, particularly testosterone, possess known immunomodulatory effects. Although congenital hypogonadotropic hypogonadism (CHH) differs in pathogenesis from classic hypergonadotropic hypogonadism (e.g., Klinefelter syndrome), exploring the potential impact of a hypogonadal state on the immune environment is intriguing. For instance, Panimolle et al. (2021) found a significantly higher prevalence of non-organ-specific autoantibodies (e.g., antinuclear antibodies) in adult men with 47,XXY Klinefelter syndrome (a form of hypergonadotropic hypogonadism) compared to healthy controls. That study suggests that X chromosome dosage effects and the endocrine environment resulting from long-term hypogonadism may collectively contribute to a predisposition for immune dysregulation.

Although the core defect in CHH lies in insufficient GnRH secretion, and our cohort did not assess immune-related markers, the aforementioned study raises a biologically interesting question for future exploration: In CHH patients, could the long-term hypogonadal state resulting from pre-treatment HPG axis dysfunction also impart some influence on the immune microenvironment (e.g., testicular local immune cell function or systemic mild immune skewing)? And could such an immune status, in turn, indirectly affect the responsiveness of testicular Leydig and Sertoli cells to exogenous gonadotropin stimulation? This remains hypothetical at present. Future studies integrating immune-related biomarkers (e.g., cytokine profiles, immune cell subsets) could help to more comprehensively dissect the mechanisms underlying the heterogeneity of CHH treatment response from the higher dimension of the ‘neuroendocrine-immune network,’ thereby opening new avenues for personalized therapy.

## 5 Conclusion

This study successfully developed an efficient and stable predictive model for CHH treatment outcomes. Methodologically, the model not only demonstrates the value of machine learning in rare disease studies with small sample sizes but also systematically reveals the physiological basis influencing treatment response through key feature clusters (cryptorchidism, genital development status, baseline gonadotropin levels) identified across multiple developmental stages—spanning the fetal period, infancy period, and prepubertal stage. Clinically, the research outcomes provide clinicians with a quantifiable tool for implementing risk stratification and personalized diagnostic and therapeutic decision-making in CHH adolescent male patients. Future studies are needed to validate and translate these findings into clinical practice.

## Data Availability

The original contributions presented in the study are included in the article/supplementary material, further inquiries can be directed to the corresponding author.
